# Companion Plants for Aphid Pest Management

**DOI:** 10.3390/insects8040112

**Published:** 2017-10-20

**Authors:** Refka Ben-Issa, Laurent Gomez, Hélène Gautier

**Affiliations:** Institut National de Recherche Agronomique (INRA), Plantes et Systèmes de Culture Horticoles (PSH), Avignon, 228 Route de l’Aérodrome, Domaine St Paul, Site Agroparc, CS 40 509, F84914, 84140 Avignon CEDEX 9, France; ben-issa-refka@hotmail.fr (R.B.-I.); laurent.gomez@inra.fr (L.G.)

**Keywords:** aphid, companion plant, intercropping, trap plant, natural enemies, repellent, volatiles

## Abstract

A potential strategy for controlling pests is through the use of “companion plants” within a crop system. This strategy has been used in several trials to fight against a major crop insect pest: the aphid. We reviewed the literature to highlight the major mechanisms by which a companion plant may act. Trials carried out under laboratory or field conditions revealed that companion plants operate through several mechanisms. A companion plant may be associated with a target crop for various reasons. Firstly, it can attract aphids and draw them away from their host plants. Secondly, it can alter the recognition of the host plant. This effect is mostly attributed to companion plant volatiles since they disturb the aphid host plant location, and additionally they may react chemically and physiologically with the host plant, making it an unsuitable host for aphids. Thirdly, it can attract natural enemies by providing shelter and food resources. In this review, the feasibility of using companion plants is discussed. We conclude that many factors need to be taken into account for a successful companion plant strategy. For the best long-term results, companion plant strategies have to be combined with other alternative approaches against aphids.

## 1. Introduction

Aphids (Hemiptera: Aphididae) are among the most destructive insect pests in cultivated plants worldwide [1,2,3]. Due to their asexual and sexual reproduction, they are capable of an extremely rapid increase in numbers [4,5]. In addition, these insects can transmit viruses [6]. The damage they cause can be very significant, and cause real economic problems for producers since crops become unsuitable for consumption [7,8]. Nowadays, chemical control based on systemic aphicides is intensively used, and ensures effective control of aphid populations. However, it is known that use of these pesticides results in major environmental and human costs. Indeed, their repeated use has increased pest resistance [9,10], raised the levels of residues in harvested products [11], and polluted both the soil and air [12]. These factors have led to increasing interest in the conception of agroecosystems less dependent on agrochemicals. Different alternative strategies for the control of aphids have been proposed in relation to plant physiology (i.e., increases in host plant resistance) and insect life cycle (i.e., conservation biological control) [13]. Intercropping is one pest-management alternative used to control pests. Intercropping is defined as “the cultivation of at least two plant species simultaneously in the same field [14] without necessarily being sown and/or harvested at the same time” [15]. Many types of intercropping have been identified based on spatial and temporal overlap of plant species, and depend on the associated crop and their evaluation after harvest [16].

For many decades, farmers have used intercropping to preserve biodiversity within agricultural fields [17,18] and establish polycultures that include two or more different crop varieties or species within the same field [19]. Intercropping is an ancient and traditional technique that has been used to provide beneficial biological interactions between crops [17,20,21] and generate different agro-environmental services [22,23], among them the protection of plants against insects [24]. Different categorical terms have been used to describe plants involved in intercropping, namely secondary plants, cover crops, and intercrops, among other examples. In this review, we use the term “companion plant” (CP) as a general term to differentiate this type of plant (or crop) from the target crop (also called the primary crop or host plant (HP)). Companion plants have the potential to protect the target crop against insects when growing nearby. CP within crops are used to intercrop at a small scale (field scale) [20,25] and the protection of the target crop against herbivores is conferred by the surrounding CP [19,26].

Knowledge of these practices has been accumulated through observations and is founded on the trial/error strategy over a long historical period [27,28]. Although the effects of CP on pests have been largely covered in the literature, the most comprehensive reviews are still very general [19,26]. Indeed, the effects of a CP differ from one species to another. Also, their mechanisms have not been clearly explained. These reviews have mainly focused on the requirements of the pest’s natural enemies. There is further potential to optimize this type of system for improved integrated pest management. As aphids represent one of the most important pests worldwide, understanding the potential of using CP in intercropping systems against aphids may be of crucial importance.

There are various reasons as to why associating companion plants with crops might result in less aphid-caused damage and therefore decrease the need for the application of pesticides. First, a CP may attract and draw pests away from the target crop. These plants, called trap plants, are more attractive to the pest, and this may divert them from their HP [29,30]. Then, by emitting volatile organic compounds (VOCs), certain CP can act either directly on aphids, by diverting their location from the target plant by repellence or masking host odors [31], or indirectly, by changing some traits of the host plant and making them unsuitable for aphids [32]. Others can act on natural enemies by providing shelter and food [33,34,35], enhancing their abundance and strengthening their rates of predation or parasitism.

The present review focuses on the potential of using CP as a tool to reduce aphid infestation. For this purpose, we first define the different categories of CP used, then expose how CP have been used in case trials and their possible mechanisms of action, and finally evaluate the efficacy of using CP in cropping systems.

## 2. Trap Plants

Trap plants are stands of plants that attract further pests and may keep them away from the main crop [36,37,38]. The use of trap plants in association with crops has been known for centuries to protect crops from insect attack, and this method has been exploited in many traditional farming systems [30,39,40]. The use of these plants in cropping systems is based on the fact that insects show a marked preference for certain plant organs, cultivars, species, or phenological stages [29]. Hence, trap plants are more attractive to the pest and easier to find than the host plants. Trap crops can be plants or part of plants (buds, flowers) [29] that serve as a temporal host crop. Therefore, trap crops have been included in the concept of associated crops and pest management. Hokkanen [37] defined trap plants as “plant stands grown to attract insects or other organisms like nematodes to protect target crops from pest attack, preventing the pest from reaching the crops or concentrating them in a certain part of the field where they can be economically destroyed”. This definition was later broadened by Shelton and Badenes–Perez [39] as “plant stands that are per se or via manipulation, deployed to attract, divert intercept, and/or retain target insects or the pathogens they vector in order to reduce damage to the main crop”. Shelton and Badenes–Perez [39] found that the efficacy of specific trap crops depends on their inherent characteristics, the value of the target crop, the spatial and temporal characteristic of each, the behavior and movement patterns of insect pests, and the agronomic and economic requirements of the production system.

Trap cropping is a method for manipulating pest behavior [29,41,42] and managing pests, but also for enhancing predator and parasitoid populations [30,43]. A trap crop can be of just one species or a combination of multiple plant species [38]. According to Hurej [30], trap crops must have some specific characteristics: they must occupy a small space, they usually need to be planted earlier than the main crop, and they need to be more attractive to the pests than the primary crop [29]. In many instances, a trap crop has been used in a push–pull strategy (the so-called stimulo-deterrent diversionary (SDD)) which requires the intervention of another component such as semiochemicals [44]. Indeed, semiochemicals stimulate pests and push them away from the crop; at the same time, the pests are attracted towards a non-valued resource or trap crop [42]. Foster and Harris [41] found that the SDD strategy is efficient mainly during the first few weeks following its application. In addition, the SDD strategy is deployed to attract predators or parasitoids into the crop. In the case of aphids, the attraction of parasitoids at an early stage of population development is achieved by the use of synomones released by plants [42] or pheromones as attractants [37]. Trap crops are considered as “decoys”, which can (1) cause the mortality of insects due to the production of toxin, or (2) reduce fecundity due to a lower resource or the imbalance of some essential nutritional materials [45]. Trap crops are used also to apply the strategy of the “attract–annihilate”. According to Foster and Harris [41], this strategy is a behavioral manipulation method which consists of attracting pests to a site and then removing them from the environment. The efficacy of trap crops can be further enhanced when these plants are “dead-end” trap crops, i.e., that are highly attractive to pests but upon which their offspring cannot survive [46].

The technique of trap cropping showed high effectiveness against several insects [47], which were mainly brassica pests [38] (*Plutella xylostella* (Lepidoptera: Plutellidae) [48], and *Delia radicum* (Diptera:Anthomyiidae) [49]). However, trap cropping is not overly developed for aphids because their host selection is often considered as a passive process that mainly depends on wind [50,51]. Potting et al. [50] concluded that small insects such as whiteflies, mites, and aphids have limited ability to detect their hosts. They suggested that trap crops act as a barrier when they are taller than the main crop and planted in the borders.

In some cases, the importance of trap crops in decreasing aphid proliferation and viral transmission has been demonstrated. For example, Hussein and Samad [52] reviewed field trials conducted in Malaysia with respect to the effectiveness of intercropping chili (*Capsicum annuum*) plants with maize (*Zea mays*) or brinjal (*Solanum melongena*) as a trap crop. The results showed that populations of *Aphis gossypii* found on chili plants were significantly reduced in plots where chili was grown with a trap plant compared to the control (monocrop) and consequently limited damage caused by the chili veinal mottle virus. Difonzo et al. [53] also showed that using soybean (*Glycine max*), sorghum (*Sorghum bicolor*), and winter wheat (*Triticum aestivum*) as trap plants reduced potato virus Y incidence in seed potato (*Solanum tuberosum*), which is transmitted by various aphid species.

## 3. Companion Plants Altering Host Plant Selection

Search behavior and the selection of vital resources for insects such as aphids can be divided into three steps: habitat location, host location, and host acceptance [54,55]. During these three steps, positive and negative external stimuli interact also with the internal factors of the insect, allowing for the acceptance or rejection of the HP [55]. Generally, visual (colors, contrasts) and olfactory stimuli are involved in aphid host-finding [56,57]. Olfaction is typically the major sensory modality during the first two steps, while chemoreception dominates the third [55]. Some CP are known to release chemicals (Table 1) that may affect the movement, feeding, and reproductive behavior of aphids. Strategies using this type of CP have been developed to disturb the process of HP selection either directly on aphids by affecting their habitat and host selection (masking, repellency), or indirectly by modifying HP acceptability by aphids. Table 2 lists examples of studies that evaluated the altering effect of CP on HP.

### 3.1. Plants Masking Host Plant Odors

CP odors can mask HP odor and make them unsuitable resources. This concept was firstly developed by Tahvavainen and Root [71] as “associational resistance”, and was then supported by other researchers [72,73]. Different forms of action on aphids, on HP, or even on volatiles released by HP have been proposed to explain it. While many studies using chemicals confirm this potential masking effect of non-aphid HP [74,75,76], only a few studies were run with living CP (Table 2). According to Visser and Avé [77], masking is “a disturbance of the attractive complex by artificially changing the relative proportion of the components”. Therefore, the insects receive different chemical information and cannot locate their HP. The masking effect results from the inhibition of the locomotive aphid movements toward an attractant source [78]. Therefore, volatiles emitted by CP are masking substances that reduce the ability of aphids to find their HP.

Mechanisms which explain the interference between host and non-HP are not well known but have been explained, in many cases, by a neutralization of the behavioral responses using repellent compounds [79]. A study on the onion aphid *Neotoxoptera formosana* suggests that odors of host plants *Allium fistulosum* and *Allium tuberosum* in olfactory tests were non-attractive when they were combined with α-pinene from rosemary oil (*Rosmarinus officinalis*), which masked HP profile and disturbed the behavioral response of this aphid [80]. This single compound, tested alone, was repellent to the aphid. Nottingham et al. [81] reported analogous findings after studying the response of the black bean aphid (*Aphis fabae*) to HP odor. The 3-butenyl or 4-pentenyl isothiocyanate emitted from savory (*Satureja hortensis*) and thyme (*Thymus vulgaris*) had a masking effect with respect to attractive host odor, but when tested alone it was repellent to the black bean aphid. Other compounds that did not elicit any behavioral response when presented alone have also been shown to mask HP odor [73,78]. Thus, a compound which per se cannot induce a behavioral response may provide reduced recognition of an HP in a blend. These studies strengthen the potential of CP odors to modify aphid behavior.

Works with whole living CP are rare. Amarawardana et al. [60] proposed a test in a cage chamber to demonstrate how whole CP odor masks host odor and affects green peach aphid (*Myzus persicae*) behavior. The study showed that *M. persicae* is normally attracted by their HP (sweet pepper, *Capsicum annuum*) and repelled by the odor of chives (*Allium schoenoprasum*). When the odors of the two plants are blended, aphids were neither attracted nor repelled by their plant host. However, five days later, the sweet pepper alone became repellent to the aphids. Authors suggested that the odor of chives adheres on the leaves of sweet pepper, masking its odor and preventing sweet pepper identification by the aphid.

The simplest explanation is an interaction between the CP and HP that generates an odor blend unrecognizable by aphids. In fact, the process is more complex and the odor perception by aphids and their behavioral response may depend on the nature, the quantity, and the proportion of components present in the mixture. For example, Zaka et al. [82] found that the response to odor was dose-dependent, and the dose of VOCs had to be increased to achieve an effective level against pests. Thus, the presence of a repellent volatile in a CP profile does not guarantee that this plant is effective [83], as the effect of a volatile compound may change with its concentration, and its effect may be influenced by other compounds via synergy, suppression (masking effect), or hypoadditivity (the more attractive effect of HP) [84].

### 3.2. Plants Releasing Repellent Volatiles

Repellent plants are defined as neighboring non-HP that disrupt insect behavior and avoid feeding activity on HP. In many cases, the repellent effect of CP was mentioned to explain lower aphid colonization on their hosts [85]. However, CP repellency has not been experimentally supported. Indeed, Finch and Collier [54,86] developed a biological approach based on the hypothesis that insects only react to volatiles emitted by their HP; those from non-HP are chemically neutral. Hence, they believed that using CP is only effective when insects land on them (inappropriate landings) and suggested that it is their green leaves and not their odor that disrupt insect behavior [25]. This theory was not supported by other reports, which showed that insects can detect and distinguish host and non-host odors to manage their movement. For example, Visser et al. [87] tested 35 volatiles of plants to detect the reception of aphids as well as their relative reaction. Results showed that electroantennogram responses differed between aphid species. Also, laboratory assays found that aphids can individually select their HP from a wide range of non-host vegetation [88].

To investigate the repellent effect of CP on aphids, various experiments have been performed with plant extracts or essential oils, in field and lab studies [80,81,89,90]. The efficiency of VOCs liberated by chemical CP extracts was studied on several species of aphids, including generalists and specialists [81]. Indeed, volatiles affect the direction and the behavior of winged and wingless aphids before contact with the HP [91,92,93]. Laboratory tests have shown that isothiocyanates and myrtenal, which are compounds emitted by the Brassicaceae family, are repellent for the black bean aphid (*A. fabae*) [74]. These compounds can be used by aphids to identify and avoid a wide range of non-HP and ecosystems where their hosts are unlikely to be present [94]. Also, we found that many terpenoids such as α-pinene, 1,8 cineole, or camphor in VOCs emitted by CP (i.e., rosemary) [83] have a repellent effect on aphids [80,95]. Starting from the assumption that VOCs emitted by the whole living plant or by their chemical extracts have similar properties, we believe, in agreement with Kogel et al. [96], that repellent plants can be used to avoid the colonization of aphids and protect the main crops.

Recently, evidence of the role of VOCs from living CP was provided by olfactory tests [95,97]. In the field, Lai et al. [62] conducted an experiment over two years by planting white garlic (*Allium sativum*) one month before transplanting tobacco plant (*Nicotiana tabacum*). The appearance of winged green peach aphids (*M. persicae*) was delayed in the host crop and their abundance was decreased even during the aphid peak period, thereby reducing tobacco mosaic virus transmission as compared with monoculture. Authors suggested a repellent effect of garlic VOCs on aphids. When the habitat contains repellent volatiles, these may rescind host location and require aphids to change their orientation. Similar effects were obtained with mustard aphids (*Lipaphis erysimi*) by intercropping mustard (*Brassica napus*) with onion (*Allium cepa*) and garlic (*A. sativium*) in different ratios [21]. The strong aphid-repelling action of the *Allium* spp. is well established [60,63,65,98]. It could be connected with the presence of high-sulfur compounds (94%) [99]. These compounds are degradation products of alkylsulfate, which are known for their protective potential against storage insects. Similarly, Liliaceae plants produce many allelochemical volatiles used to control pests. The odors of these plants play an important role in aphid behavior and breeding [88,100].

In several studies, the choice of CP was often based on their essential oils’ repellence properties. For example, intercropping CP in pear orchards (*Pyrus betulaefolia*) was tested by Beizhou et al. [68] with three aromatic plants: summer savory (*S. hortensis*), ageratum (*Ageratum houstonianum*), and basil (*Ocimum basilicum*), with beneficial results. A significantly lower population of major pests, especially *Aphis citricola*, as compared with natural grasses or clean tillage, supported the repellent chemical effect of these aromatics plants. Basedow et al. [67] found that an intercropping system of *Vicia faba* with two Lamiaceae (*O. basilicum* and *S. hortensis*) in a wind tunnel, greenhouse, or field significantly decreased the *A. fabae* population. However, it is difficult to predict a repellent effect under field conditions where additional variables and different environmental conditions may affect aphid behavior. Thus, different tests have been developed indoors under controlled conditions to help choose a CP for its repellent effect. For instance, tests carried out by Nottingham et al. using a linear track olfactometer [81] and a flight chamber [101] demonstrated the repellent effect of *S. hortensis* and *Tanacetum vulgare*, respectively, on the black bean aphid (*A. fabae*).

### 3.3. Plants Modifying Host Plant Acceptance 

Some CP disturb HP acceptance by changing their biochemistry and consequently their aphid hospitality. To better understand the mechanisms involved, we will firstly highlight how an HP can capture and decode information from neighboring plants.

Like insects that use chemical signals emitted by plants as information, plants can also detect and use chemical signals [102]. Plants have evolved complex mechanisms to communicate with their environment [103]. Interactions between below-ground or above-ground plants without physical contact is named allelopathy, which is defined as the “direct or indirect harmful or beneficial effects of a plant on another plant, through the release of compounds that escape into the environment” [104,105]. In spite of the negative effects of allelopathy on the inhibition of seed germination and shoot and root growth [106,107,108], the ecological role of allelopathy in agroecosystems generally remains beneficial for plant survival [70,109,110,111]. Plants can interpret volatiles information and use them to adapt to environmental conditions through morphological and physiological changes [112]. These mechanisms are based on the ability of plants to exchange gases. Plants can emit a mixture of volatiles that have distinct biological functions related to either plant–insect, plant–pathogen, or plant–plant communication [32]. However, the mechanisms involved have not yet been studied well enough and remain poorly understood. Plant responses can be either innate or induced [113], and vary with the type of stimulus perception. The activation of various metabolic pathways could be involved in explaining plant response, including VOC emission [114], gene expression [115], phenolic compound synthesis [116], proteinase inhibitor synthesis [117], and floral nectar production [118].

It is known that CP can enhance the response of HP and make them unattractive resources for pests [119]. This phenomenon does not consist of masking HP odor as mentioned above, but making the HP unsuitable for pests. Several studies have shown that HP can detect chemical signals emitted by neighboring plants [120], triggering their active VOC emission [114] or changes in their chemical composition [70].

During the last phase of HP selection, most insects and particularly aphids evaluate the suitability of their host. The acceptance depends on the presence of positive stimulus [74,121], but when an HP undergoes changes it could become unsuitable and thus the insect would go away [74]. Generally, specialist aphids have a rapid response for host acceptance or rejection, unlike generalist aphids, which respond rather to a large range of metabolites [122]. As defined in Table 1, deterrents that inhibit insect feeding or oviposition are normally perceived after landing on an HP [59].

Several mechanisms might explain how a CP can transform an HP into an unsuitable food source. Indeed, as the HP can emit repellent VOCs [121], generally in small quantities [102,113,123], VOC production might be enhanced by the presence of CP. This latter type of plant has the capacity to produce molecules like ethylene, methyl jasmonate, and cis-jasmone, usually used by damaged plants to prevent their neighbors from an insect attack [32,117]. Thus, it could be a means for the CP to trigger the defense of the HP against herbivores. For example, Karban and colleagues [124] showed that tobacco plants have a high level of polyphenol oxidase when intercropped with clipped sage brush (*Artemisia tridentata*), which affected the occurrence of insects. This could be due to the presence of methyl jasmonate in sage brush leaves [117], as methyl jasmonate induces proteinase inhibitor accumulation in tomato plants. In several cases, methyl jasmonate has been used as commercial substance to increase the emission of several defensive VOCs in HP, such as 6-Methyl-5-hepten-2-one in sweet pepper [125], (E)-b-ocimene in beans [126], or hydroxamic acids in wheat [127].

In addition, substances emitted from CP may be absorbed by HP roots so that HP exhibit physical and chemical responses, releasing active substances by roots or aerial parts into the atmosphere [128]. Barley (*Hordeum vulgare*) has often been used as a plant model to study direct or indirect biochemical interactions with neighboring plants. For example, laboratory tests [129] showed that barley plants become less attractive to *Rhopalosiphum padi* after exposure to exudates of quack grass (*Elytrigia repens*). Results do not distinguish a repellent or deterrent effect. The lower attraction is caused by a symbiotic interaction between microorganisms and the rhizospheres of barley plants. Glinwood et al. [70] demonstrated in chambers and with olfactometer tests that barley exposed to VOCs emitted by *Cirsium vulgare* for five days became less attractive to cereal aphids (*R. padi*). They suggested that VOCs adhered to the barley leaves and directly repelled aphids. However, olfactory tests did not detect statistically significant responses of aphids to the odor of *C. vulgare*. A possible scenario is that volatiles from *C. vulgare* induced barley to produce specific phytochemicals responses that reduced the nutritional quality of barley and therefore affected *R. padi* settlement. A similar deterrent effect, i.e., a reduced growth rate of aphid nymphs, was observed in laboratory and field conditions with intercropping barley and *Chenopodium album* [130]. A competition between the neighboring plants could explain this chemical change in barley.

Other mechanisms which may explain the unsuitability of an HP is that HP could absorb and release the VOC emitted by CP. To prove this mechanism, Himanen et al. [131] used as a CP *Rhododendron tomentosum*, a plant characterized by a very aromatic profile and a very high emission rate of terpenoids (which have repellent properties). The main VOCs released are sesquiterpenes and monoterpenes. Choh et al. [114] examined the intercropping of *Batula* spp. with *R. tomentosum* in natural conditions. Results showed that *Batula* spp. emitted large quantities of new VOCs including palutrol, ledol, and ledene even after 10 min of association. These compounds remained detectable at a significant level for up to four hours, resulting in reduced incidence of the aphid population (*Euceraphis betulae*). Authors suggested an adsorption of volatiles phenomenon, since *Batula* spp. alone is unable to produce these compounds.

Only a limited number of companion plant trials have been used to explore aerial allelopathy between plants. In a previous study, we tested the effect of 12 CP with respect to the performance of *M. persicae* on their host in growth chambers [83]. We observed that aphid performance was affected by the presence of some CP (*R. officinalis, Tagetes patula nana*) when placed close to pepper plants (*C. annuum*). According to our data, under controlled conditions and without any physical contact, VOCs from CP can disturb settling behavior and affect the performance of *M. persicae*. We also showed, under greenhouse conditions [64], a reduction in the number of nymphs in pepper plants neighboring lavender (*Lavandula latifolia*) compared with the pepper plant alone. Indeed, females may find that a site is uninhabitable for their offspring and change their reproductive strategy [132]. We suggested that sweet pepper plants became less attractive as a result of the interaction with VOCs from lavender. 

## 4. Companion Plants Attracting Natural Enemies

Some CP can attract and provide shelter and resources for natural enemies in order to improve and sustain biological pest control. The role of natural enemies to control pests has been underlined in several agroecosystems in the context of environmental management and preservation of biodiversity [12,133,134,135]. It is important to note that aphids have a diverse range of natural enemies, including predators and parasitoids (for example, Syrphidae, Coccinellidae, Chrysopidae, Miridae, Aphelinidae, Braconidae, and Carabidae). Despite their undeniable effectiveness, sometimes they cannot stop the exponential growth of aphid populations [136,137,138], probably due to several factors that limit their abundance (for example pesticides and lack of food sources and HP) [139]. In addition, agricultural systems do not provide the necessary resources throughout the season. Two approaches can promote the use of natural enemies: importing natural enemies or conserving the natural enemies of the pests [140]. Van Lenteren [141] found that the introduction of natural enemies in agricultural systems must be regular and sufficient. Also, imported natural enemies need time to adapt to the environment. Conservation biological control depends upon naturally occurring enemies of the pests [140]. The abundance and diversity of natural enemies increases in response to a variety of conservation measures, including plant and habitat diversification [142]. Different approaches have shown that the installation of CP increased and favored the development of these organisms, which promoted a more sustainable control of pest populations [33,68,139,143]. Many studies suggested that CP can represent a valued resource of pollen and floral and extra floral nectar [144,145], as well as improve the availability of resources necessary for optimal performance of natural enemies, maintaining their survival, fecundity, and longevity [34,146]. In agricultural systems, the spraying of food as nectar and pollen was used as a food supplement, while the establishment of flowering plants can provide even more stable sources throughout the season [147]. 

Several experiments evaluated the potential of flowering plants to preserve and attract natural enemies of aphids to the main crop. These plants are known to produce large quantities of easily accessible food resources. For example, sesame (*Sesamum indicum*) has shown potential in the laboratory as a nectar plant to enhance biological control in Asian rice systems [148]. Bugg et al. [144] used hairy vetch (*Vicia villosa*) as a cool season cover crop, which has been observed to increase the numbers of predatory lady beetles in pecan orchards. White et al. [149], reported that *Phacelia tanacetifolia* represents an important pollen resource that attracts hoverflies and consequently reduces infestation by the aphids *Brevicoryne brassicae* and *M. persicae* in cabbage crops. In field experiments, Colley et al. [150] tested 11 flowering plants intercropped with broccoli to enhance the biological control of aphids. As a result, flowering plants increased the number of hoverflies and parasitic wasps in alyssum plots and the number of hoverfly eggs laid on the broccoli plants, as compared to the monoculture. However, results have not shown evidence of aphid reduction. The relationship between CP, target pests, and their natural enemies are complicated and may show variable success. Nevertheless, a lower parasitism of aphids (*M. persicae* and *B. brassicae*) by *Diaeretiella rapae* (Hymenoptera: Braconidae) was found when the broccoli crop was grown with living mulches [151]. Authors suggest that plant diversity can provide a barrier limiting parasitoid searching efficiency. In addition, the attractiveness of CP may depend on season, species, and site. In New Zealand, for example, coriander (*Coriandrum sativum*) as a CP intercropped with cabbage has been shown to attract adult hoverflies and decrease the aphid number only early in the season. Indeed, the flowering of CP needs to coincide with the activity of natural enemies [152]. Morris and Li [153] detected a higher number of hoverflies in coriander in New Zealand as compared to Japan, where the flowering time of coriander is shorter. Martínez-Uña et al. [154] showed that *Calendula arvensis* and *C. sativum* were the most visited species by hoverflies. *C. arvensis* received a high number of visits throughout a long period, whereas the visits to *C. sativum* were concentrated in a short blooming period. Indeed, some natural enemies are considered specialized feeders. For example, Haslett [155] found that certain hoverfly species are highly selective in their diets, while others are generalist in their foraging ability.

In addition to food resources, visual and olfactory cues should be considered in attracting natural enemies in the targeted area. Some colors may attract natural enemies more so than others. For example, in flight chamber experiments, *Cotesia rubecula* (Hymenoptera: Braconidae) was shown to spend more time searching in yellow targets [156]. Visual preference tests were in occurrence with these results, showing that the Asian ladybird *Harmonia axyridis* (Coleoptera: Coccinellidae) clearly prefers yellow to white, blue, red, orange, and green [157].

CP can release certain volatiles that attract natural enemies. These volatiles may affect natural enemies’ movements and guide them to find their prey habitats [158,159,160]. For example, Schaller and Nentwig [161] found that ladybirds (*Coccinella septempunctata* (Coleoptera: Coccinellidae)) were attracted by the odors of beret extracts (*Berberis vulgaris*) and camomil flower buds (*Tripleurospermum inodorum*). However, the same volatiles may be used both by natural enemies and herbivores in their search for food resources [160].

Aromatic plants have also been used as CP to enhance the abundance of natural enemies. For example, in a pear orchard ecosystem, Beizhou et al. [68] found that using aromatic plants such as summer savory (*S. hortensis*), ageratum (*A. houstonianum*), and basil (*O. basilicum*) between pear tree rows enhanced the activity of the *C. septempunctata* and reduced the incidence of *Aphis citricola*. Results showed that intercropping with aromatic plants contributed to the earlier appearance of natural enemies and the shorter occurrence duration of pests, mainly aphids. Other various aromatic plants (*Mentha canadensis*, *A. houstonianum*, *T. patula*, and *O. basilicum*) have also been used in apple orchards; they increased predator abundance and species richness during the aromatic plants’ flowering period [162]. 

CP can be used under different arrangements. They can be grown around or within crops to increase the density and diversity of predators and parasites [152]. For example, intercropping buckwheat between rows of peach trees increases the population density of hoverflies and also decreases aphid populations [163]. Lin et al. [164] found a high number of predators (spiders and lacewings) when cotton was bordered by alfalfa cutting areas, and attributed to this an efficient control of the cotton aphids for at least two weeks. The same findings have been reported in Australia using border lablab (*Lablab purpureus*) and lucerne (*Medicago sativa*) with cucurbit against *A. gossypii* and *M. persicae* [165].

Wildflower strips are a commonly used measure aimed at the conservation of natural enemies and pollinators [166]. Several studies have assessed the potential of sowing wildflower strips as mixtures to favor natural enemies and enhance pest control. For instance, Tschumi et al. [167] showed that the presence of tailored flower strips enhanced the abundance of key natural enemies of aphids (hoverflies, lacewings, and ladybirds) in nearby potato crops. Also, the abundance of winged and apterous aphids was significantly reduced and more adult hoverflies were found in wheat in between wildflower strips, as compared to monoculture wheat plots [168]. However, the attraction of natural enemies may depend on the use of wildflower strips. For example, no significant differences were observed in wheat tillers for adult lacewings, ladybeetles, and parasitoids [168]. The selection of wildflower strips depends on the chosen traits like the abundance and quality of nectar and pollen [169]. 

## 5. Conclusions and Perspectives

**Figure 1:**
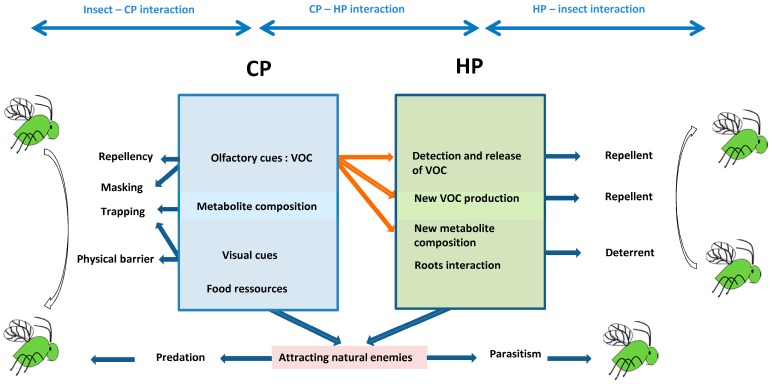
Potential interactions between companion plants, aphids, natural enemies, and host plants. Interactions and mechanisms described are based on the available literature. CP: companion plant, HP: host plant, VOC: volatile organic compound.

Using CP in intercropping with target crops is a promising alternative method to chemicals to improve aphid management. We showed that many companion planting schemes have been designed to reduce the aphid population, and several mechanisms have been considered (Figure 1). However, the choice of adapted CP remains an issue and their efficacy might not be guaranteed in all cases [24,64]. Indeed, many factors that affect the success of an intercropping system design need to be considered. For instance, intercropping CP may take several forms, and its efficiency may depend on the arrangement, the density, and the distance between CP and HP [64]. Similarly, the timing of the most effective phenological stage of CP with respect to the aphid infestation period and the aphid life cycle must to be taken into account [64]. A CP also should not offer a home for other pests per se [120], and a CP needs to be in the most appropriate phenological stage to provide shelter and food resources, especially for early natural enemies such as hoverflies [137].

On the other hand, using CP in a crop system may create constraints for farmers (for example with respect to tillage and irrigation). Therefore, the choice of CP may depend on their economic return. For example, aromatic or medicinal plants may be harvested to offset part of the costs and provide a direct income. We suggest that CP be introduced and tested at different scales (in greenhouses or orchards, for example) as they can be used without reducing the space. We have noted in our previous experiment that sweet pepper can be commercially produced under protected environments using CP [64].

Companion plants may work simultaneously, influencing both top-down (i.e., reinforcing host plant defense) and bottom-up (i.e., enhancing biological control) mechanisms. In several cases, companion plants have been shown to simultaneously have a repellent effect against pests and an attractive effect on natural enemies [135], which might be useful to enhance the functionality of CP. In addition, several push–pull systems with trap crops have been successfully combined with repellent plants. Further research is needed in order to test the potential effect of combined CP. For example, introducing flowering plants within fields may enhance the abundance of natural enemies and simultaneously create barriers to aphids. Additionally, the selection of an adequate oviposition site by natural enemies such as Syrphidae females, that is, the laying of eggs close to an aphid colony, is essential to the survival and development of their offspring [137]. In this case, the presence of trap plants near CP that attract natural enemies may be of potential interest.

We have shown that the HP location is a crucial factor in the life cycle of aphids as well as a critical element for crop protection. Indeed, the presence of CP appears to be a preventative intervention with regard to the sensitivity of the winged aphids to CP volatiles. For example, the presence of CP may prevent aphid colonization or delay their settlement during the spring and autumnal return, and consequently reduce the number of sprays needed during periods of lower aphid activity. Many laboratory trials have demonstrated the efficiency of certain CP due to their VOCs. However, under field conditions and due to the fluctuations of abiotic factors, the concentration of VOCs might not be enough to disturb aphid host selection. Optimizing VOC emission may be a solution to make them more effective. Indeed, the production of volatiles is sensitive to several environmental factors [170,171,172]. For instance, their production may be affected by water availability. A water deficit (four days) decreases the emission of sesquiterpenes from rosemary plants (*R. officinalis*) [172]. Clipping of CP can also promote VOC emission and reduce plant infestation [173]. In addition, the time of planting affects VOC emission and essential oil production [174].

It is clear that there is still much work to be done in order to optimize the services provided by the intercropping of CP in crop systems. We believe that using only a CP strategy will not completely replace chemical control or provide satisfactory control because of only partial effectiveness, which may be limited by several constraints (climatic conditions, CP density, CP phenology). However, the use of CP can be associated with other integrated pest management approaches (i.e., use of resistant host plants, spraying of extracts and essentials oils, early release of natural enemies, and use of Alt’carpo nets). 

## Figures and Tables

**Table 1:** Designation of chemicals according to the response they elicit from insects [58] and the type of receptors involved [59]. HP: host plant.

Definition	Response Elicited from Insect	Type of Receptors Involved
Repellent	A chemical which causes insects to make oriented movements away from its source (without contact with the HP)	Olfactory receptors
Arrestant	A chemical which causes insects to aggregate because it reduces their tendencies to move away from its source (without contact with the HP)	Olfactory receptors
Deterrent or feeding deterrent	A chemical which inhibits feeding, mating, or oviposition behavior after landing on the HP	Gustatory receptors

**Table 2:** Examples of studies that evaluated the altering effect of companion plants on host plants and the different mechanisms involved.

Species of Aphid	Host Plant	Companion Plants	Proposed Mechanisms	Reference
*Myzus persicae*	*Capsicum annuum*	*Allium schoenoprasum*	Masking odors Repellency Reduces the attractiveness of hosts	[60]
*Brevicoryne brassicae*	*Brassica oleracea * *Bently F1*	*Tagetes patula nana * *Calendula officinalis*	Repellency	[61]
*Myzus persicae*	*Nicotiana tabacum*	*Allium sativum*	Repellency Deterrent effect	[62]
*Myzus persicae * *Aphis gossypii*	*Solanum tuberosum*	*Allium sativum*	Repellency Reduces the attractiveness of hosts	[63]
*Myzus persicae*	*Capsicum annuum*	*Ocimum basilicum * *Rosmarinus officinalis* *Lavandula latifolia*	Repellency Reduces the attractiveness of hosts	[64]
*Brevicoryne brassicae*	*Brassica oleracea*	*Allium cepa*	Reduces host-finding ability	[65]
*Brevicoryne brassicae*	*Brassica oleracea*	*Secale cereal*	Reduces host-finding ability	[66]
*Aphis fabae*	*Vicia faba*	*Satureja hortensis * *Ocimum basilicum*	Deterrent effect Repellency	[67]
*Aphis citricola*	*Pyrus communis*	*Satureja hortensis * *Ocimum basilicum*	Repellency	[68]
*Lipaphis erysimi*	*Brassica napus*	*Allium cepa * *Allium sativum*	Repellency Deterrent effect Disrupts behavior	[21]
*Macrosiphum rosivorum*	*Rosa chinensis*	*Tagetes patula*	Repellency Allelopaty change	[69]
*Rhopalosiphuim padi*	*Hordeum vulgare*	*Cirsium* * vulgare*	Allelopaty change Reduces the attractiveness of hosts	[70]
